# Microplastics in the Marine Environment: Sources, Fates, Impacts and Microbial Degradation

**DOI:** 10.3390/toxics9020041

**Published:** 2021-02-22

**Authors:** Huirong Yang, Guanglong Chen, Jun Wang

**Affiliations:** 1 Joint Laboratory of Guangdong Province and Hong Kong Region on Marine Bioresource Conservation and Exploitation, College of Marine Sciences, South China Agricultural University, Guangzhou 510642, China; hry@scau.edu.cn (H.Y.); glchen@scau.edu.cn (G.C.); 2 Guangdong Laboratory for Lingnan Modern Agriculture, South China Agricultural University, Guangzhou 510642, China

**Keywords:** source, fate, bacterial degradation, marine environment, microplastics

## Abstract

The serious global microplastic pollution has attracted public concern in recent years. Microplastics are widely distributed in various environments and their pollution is already ubiquitous in the ocean system, which contributes to exponential concern in the past decade and different research areas. Due to their tiny size coupled with the various microbial communities in aquatic habitats capable of accumulating organic pollutants, abundant literature is available for assessing the negative impact of MPs on the physiology of marine organisms and eventually on the human health. This study summarizes the current literature on MPs in the marine environment to obtain a better knowledge about MP contamination. This review contains three sections: (1) sources and fates of MPs in the marine environment, (2) impacts of MPs on marine organisms, and (3) bacteria for the degradation of marine MPs. Some measures and efforts must be taken to solve the environmental problems caused by microplastics. The knowledge in this review will provide background information for marine microplastics studies and management strategies in future.

## 1. Introduction

Plastics have brought a lot of benefits to modern life, driving the tremendous growth in plastic demand, because of their low cost, light weight, and durable character [1,2]. It was reported that 3 billion tons of plastic were manufactured in 2016, and every year, some 8 million tons of plastics will eventually enter the marine environment [3,4]. One of the consequences of this accumulation in the marine environment is the low percentage of recycled plastics [5,6] as just 9.4 million tonnes of plastic postconsumer waste were collected in Europe to be recycled in 2018 (both inside and outside the Europe) [7]. Plastic pollution is already ubiquitous in the ocean environment. Most worrying of all, it was estimated that the weight of plastics in the ocean will be more than that of the fish by 2050 [8].

**Figure 1:**
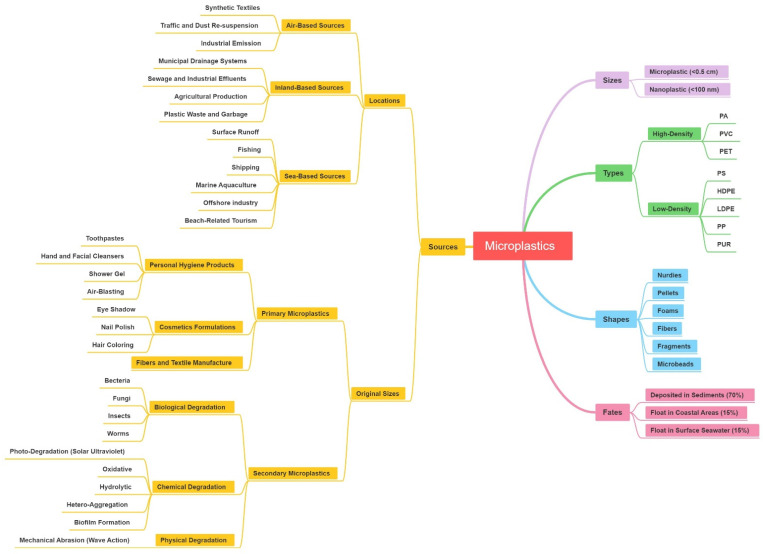
The basic characteristics of microplastic about size, type, shape, source and fate.

Microplastics (MPs) are plastic fragments or particles with a diameter of less than 5 mm formed by fragmentation of larger plastics [9,10,11,12,13,14]. Plastics can fragment into smaller particles in the marine environment [15,16]. Microplastics appear in various shapes, such as foils, foams, fibers, pellets, fragments and microbeads [17,18]. Generally, plastics are chemically diverse. The density of polyamide (PA), polyvinylchloride (PVC), and polyethylene terephthalate (PET) are higher than that of seawater, increasing the settlement rates in sediments, while polystyrene (PS), high-density polyethylene (HDPE), low-density polyethylene (LDPE), polypropylene (PP) and polyurethane (PUR) with lower densities might float mainly on seawater [19,20,21,22] (Figure 1).

**Figure 2:**
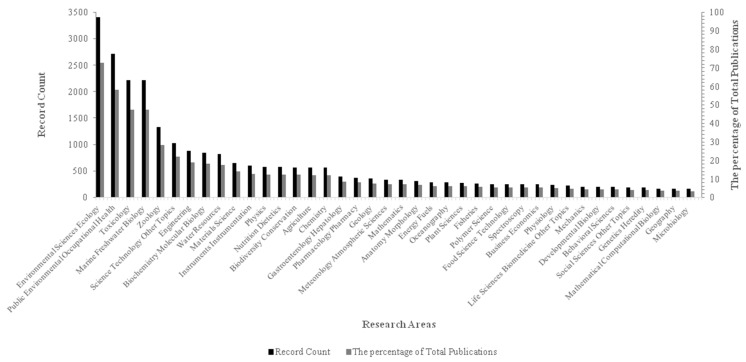
The record count and the percentage of total publications in the top 40 research areas related to the assessment of the microplastic effects on organisms and bacterial degradation over time. Source: Web of Science; Period: 1944–2020; Total Publications: 4685; h-index: 162; Average citations per item: 29.31; Sum of Times Cited: 137,315 (without self-citations: 53,749); Citing articles: 32,830 (without self-citations: 29,560). TS = (microplastic * OR micro-plastic * OR plastic particle * OR plastic particulate OR plastic debris OR plastisphere * OR microplastic pollution *) AND (source * OR fate * OR occurrence * OR distribute * OR influence * OR impact * OR affect OR risk * OR effect * OR exposure * OR exposed OR colonize OR colonization OR bacteria * OR germ * OR microbiological OR microorganisms OR microbial OR microbiota OR macrobiotic OR biotechnological OR degrade * OR degradation * OR biodegradation * OR biodegrade * OR organisms * OR creature * OR biota * OR habitat *) AND (marine * OR ocean * OR sea * OR seawater * OR beach * OR shore * OR coast * OR seacoast * OR seaboard *).

Microplastics are prevalent in the environment, especially the marine environment, due to hydrodynamic processes, transportation by wind and ocean currents, ranging from the large ocean gyres such as the Pacific Ocean [9,23], the Atlantic Ocean [24], Indian Ocean [25], polar regions [26,27,28], and the equator [29], and from coasts [30,31] to open seas [32,33]. It was estimated that more than 15 trillion microplastics were present in the global ocean in 2014, weighing more than 93 thousand metric tons [34]. MPs are abundant in the Great Pacific Garbage Patch, with about 1.69 trillion (94%) floating pieces [10] that are microplastics. Generally, microplastics pollution is already a ubiquitous presence in the ocean environment, which contributes to exponential public and scientific concern in last decade and different research areas (Figure 2).

Due to their tiny size, MPs can be ingested accidentally by marine species [35,36], such as fish [37], mussels [38,39,40], zooplankton [41], seabirds [42], sand hoppers [43] and worms [44].

The ecological threat of MPs to the oceanic environment and their health risk to organisms have not been fully clarified, but given the sharply increasing amount of evidence about the presence and effects of MPs in the marine environment, MP pollution has become a great environmental concern [45,46,47,48,49,50,51,52,53,54,55]. Some measures and efforts must be taken to solve the problems caused by microplastics and improve plastic waste management.

The present review will summarize existing research on MPs in the marine environment to provide a better understanding about MPs contamination in marine environment. This review contains three sections: (1) sources and fates of MPs in marine environment, (2) impacts of MPs on marine organisms, and (3) bacteria for the degradation of marine MPs.

## 2. Sources and Fates of MPs in Marine Environment

### 2.1. Sources of marine MPs

Marine microplastic pollution originates from a variety of sources and can generally be divided into inland-based, sea-based and air-based sources [19,56,57,58] (Figure 1). Rivers are considered to be the most important pathways for microplastics to be transported from inland areas to the ocean [59]. About 80% of the plastic pieces in the ocean originated from the terrestrial environment [12,56,60]. Plastic debris in municipal drainage systems and sewage effluents, or improper management of inland areas is blown into the sea through rivers, and plastic waste from beach-related tourism is discarded directly into the environment [18,56,57,61]. Sea-based sources originate from fishing, shipping and offshore industries [62,63]. The emissions and leaks of large shipping are considered as an important source of microplastics [64]. Loss and damage of fishing and aquaculture equipment can easily introduce plastic particles into the ocean [9,65,66]. Followed by marine aquaculture, the main offshore source is the world’s fishing fleet [67], garbage illegally discarded from ships or offshore platforms [68], and a large proportion of items comes from lost containers [56,69]. In addition, airborne MPs are also important sources [70].

According to their original sizes, microplastics can be divided into two groups. Originally designed plastic microbeads, industrially produced particles and powders (<5 mm in diameter) could enter the ocean directly through sewage effluent, which is called primary microplastics [57,71]. When subjected to the combined effects of physical, biological and chemical processes, large plastic fragments are broken down and degraded into tiny fragments, which are secondary microplastics and can be transported to the marine environment [72,73,74]. Primary microplastics are widely used in personal hygiene products containing abrasives and scrubs (like toothpastes, hand and facial cleansers; shower gels and air-blasting aids, etc.) [28,75,76,77,78], cosmetics formulations (such as eye shadow, nail polish, hair coloring, etc.) [79,80], and also fiber and textile manufacture [81].

Generally, secondary microplastics imply the breakdown of large plastic debris due to biological, chemical and physical degradation, which are representative of microbial species biodegradation, photodegradation (solar ultraviolet radiation) and mechanical abrasion (wave action), respectively. Plastic debris in the ocean are subject to mechanical damage and photodegradation well as oxidative degradation, which break down fragile plastics into microplastics [82,83]. Besides, microplastics can further degrade to nano-scale plastic pieces [40]. These microplastics and nanoplastics are more easily ingested and will have long-term adverse impacts on the marine environment, making them become a public concern in the future [40,83,84,85] (Figure 1).

### 2.2. Fates of Marine MPs

Generally, debris in any water body will ultimately enter the ocean. Transported by water power and wind power, microplastics gradually migrate and diffuse through the ocean, eventually becoming as ubiquitous as they are today, ranging from the large ocean gyres (e.g., the Pacific Ocean [9,23]; the Atlantic Ocean [24]; Indian Ocean [25]) to the polar regions and equator, from densely populated areas to remote islands, and from beaches down to the abysses of the sea [26,27,29,30,33]. They come in various shapes, with fibers being the most common form, followed by fragments. Marine circulation, estuaries and other coastal areas where humans are active are the ecosystems most seriously polluted by microplastics [86,87,88]. Approximately 70% of marine plastic debris is deposited in sediments, 15% floats in coastal areas and the remainder float on the surface seawater (Figure 1). Microplastics will be accumulated in the global ocean circulation, since some of them are less dense than seawater and float on the sea surface, and the converging sea currents concentrate and retain debris for a long time [23,35,89,90]. According to the surveys, there are only at least 7000 tonnes of plastic debris on the surface of the high seas [89], but at least 4.8 million tonnes of plastic debris enter the marine environment each year [91], which is inconsistent with data on surface plastics, suggesting that a significant number of plastics sinks to unknown depths. Microplastics have even been found on the seafloor at 2200–10,000 m depth, containing both high [92] and low [93] density (relative to seawater) microplastics. This indicates that the migration of microplastics is a dynamic process, which may not only be carried to every part of the marine through physical effects such as crushing and coastal deposition, but also through chemical processes such as oxidation or hydrolysis [62,94], and may also be carried to every part of the ocean through biological absorption, digestion and excretion [95].

Weathering processes, biodegradation processes, oxidative and hydrolytic degradation [62,93] and hetero-aggregation and biofilm formation [96,97] could significantly affect the fate of microplastic pieces in the oceanic environment (Figure 1). Biological pollution and subsequent chemical deposition of plastics, could dominate migration in seawater environments [98,99,100]. Therefore, according to biofilm growth, sedimentation and marine depth distribution of various physical factors such as light, salinity, water density, temperature, and viscosity, a theoretical predicted model was established to simulate the impact of biological pollution on the migration of microplastics, and forecast the size-dependent vertical migration of sea microplastics [101].

In addition to the origin and fate of MPs, many papers have also focused on the particle size, shape, type, color and mesh size of MPS and how to sample it to fully understand the characteristics of MPS in marine ecosystems (Table 1). This information will be helpful for further evaluation of plastic production plans and for more scientific and effective control of plastic products [102,103,104,105,106,107,108,109,110,111,112,113,114,115,116,117].

## 3. Impacts on Marine Organisms of MPs

Recently, abundant literature has assessed the accumulation of microplastics in marine organisms through direct contact [36] or food chain exposure [37] to MPs. MPs are ingested by organisms and have negative effects on their development, metabolism, reproduction and cellular response, and so on [118,119,120,121,122,123,124,125,126,127,128,129,130,131,132,133,134].

### 3.1. Exposure

Basically, there are two primary modes of MP exposure for marine organisms: bathing contact and ingestion. Bathing, of course, is the most common contact method in MP bioassays of natural marine environments, making it possible to study the various adverse effects caused by microplastics on the aquatic organisms through contact [36]. For example, microplastics could attach to the surface of skin, crust and ectoderm of *Artemia franciscana* [55]. Besides, microplastics could be ingested by low-nutrient organisms (like zooplankton such as artemia [55,118,135] and larvae of various marine animals such as shellfish and sea squirts [118,135,136], which are more readily available and easily exposed to suspended microplastics, since microplastics are similar than planktonic organisms and sediments in size and density [38,55,137,138,139].

### 3.2. Translocation

Microplastics are found in the circulatory system and tissues of some marine organisms because they could pass through epithelial tissues and even cell membranes. This phenomenon was called “translocation” [36,140]. For example, after a 3 h exposure, HDPE was detected in mussels’ stomachs and accumulated in the lysosomal system [39]. Since microplastics cannot be digested or absorbed, they can pass through cell membranes, transport through the inner layer of intestinal epithelium into the circulatory system and enter tissues after ingestion [38,56]. Therefore, MPs could be translocated and accumulated in cells and specialized tissues, such as gills and guts [141], liver [142], lysosomal system and hemolymph in blood cells [39].

Translocation efficiency depends mainly on the size of the MPs, but is also biologically affected by other factors, such as shape, concentration and the related organisms [143,144]. MP < 10 μm may be compatible with the use of membrane surface recognition elements through the epithelium [145]. As the size of microplastics decreases, the ability for microplastics to accumulate in marine organisms may increase, because the smaller the microplastics, the easier their transport. Currently, one of the main techniques for studying translocation is to expose organisms to fluorescently labeled plastic particles and then use a microscope (e.g., fluorescence and confocal microscopy) to observe MPs in the tissue, as well as do the quantitative analysis through flow cytometry [146,147].

### 3.3. Bioaccumulation and Bioavailability

The two important indexes to access the impacts of MPs to organisms are bioaccumulation and bioavailability [36]. There are interactions between MPs and organisms in the marine environment [148]. Microplastics can be ingested directly by marine organisms or transferred and accumulated in the food web from lower trophic organisms to higher trophic organisms, and the higher the trophic level, the more microplastics may be enriched in the organism [149]. In addition, toxic pollutants could be transported and accumulated in organisms along with microplastics through the ingestion, which has been demonstrated during experimental exposure tests. It has been speculated that POPs could be significantly bioaccumulated in the food web via microplastics [137,150,151].

The bioaccumulation of MPs has been identified in the digestive tract such as the oral area [33], gastrointestinal tract [37,116,142,152] and liver [153] of marine organisms, and followed by translocation to the circulatory system, other specific tissues and cells [39,141,142]. According to Bottari et al., fibrous microplastics are found in the digestive systems of *Zeus faber* and *Lepidopus caudatus* [152]. Microplastics have been reported to be found in fish populations at the bottom of the Mediterranean, with PE accounting for the largest proportion [153]. Furthermore, it has been reported that when *Dicentrarchus labrax* ingest microplastics, the particles accumulate in the liver, accompanied by oxidative stress [154]. Even some endangered species, such as bluefin tuna, have been found to have microplastics in their bodies, which raises concerns about the extent of microplastics pollution in marine species [142].

Bioavailability strongly relies on the physiochemical properties of microplastics, like their size, shape, and density [11,138]. The conclusion is that the size of microplastics is the most important factor. As the size decreases, the potential of bioaccumulation and bioavailability increase [9,138], because microplastics with smaller size are similar to planktonic organisms, and could be easily mistakenly ingested by zooplankton [36]. The irregular shape of plastic particles or fibers results in different bioavailability [155].

Additionally, biological factors could increase the microplastic bioavailability. MPs egested within fecal matter might be ingested by subsequent detritivores and suspension feeders [156], then be cast up on the benthos, attracted to the sediment, and MPs could be available for infauna, sediment-dwelling organisms capable of bioturbation [30,57,137]. Furthermore, their bioavailability in the water column is also influenced by biological fouling and aggregation, and after decontamination, they float at the sea-air interface [56] or sink below the marine surface, due to reduced buoyancy [96].

Microplastics could enhance the bioavailability of adsorbed pollutants, which has attracted more interest from scientists [135,136]. Unfortunately, due to the very high number of possible interaction factors, including physical (e.g., salinity, pH, and temperature), chemical (e.g., hydrolysis, oxidation, reduction and enrichment) and biological factors (e.g., organisms variables), it is difficult to assess how the bioavailability of pollutants enhanced by microplastics [136].

### 3.4. Toxic Effects

Microplastics have toxic effects on marine organisms. Different types and sizes of microplastics have different toxic effects on marine species, which are ultimately reflected in the physiological response of organisms and the damage they are subjected to [118,119,120,121,122,123,124,125,126,127,128,129,130,131,132,133,134] (Table 2). In addition, different microplastics also adsorb different pollutants, which combine to further damage the health of living marine organisms [150,157,158,159,160,161,162] (Table 2).

#### 3.4.1. Physiological Impacts

Some morphological changes were detected in the marine phytoplankton when they ingested microplastics. For example, some thylakoids were deformed and cell walls were thickened [118], algae homo-aggregation and algae-microplastics hetero-aggregation [118], as well as expression of certain chloroplast genes was reduced [119].

As for the development, studies examining the impact of MPs have reported significant effects on the development of marine zooplankton and other invertebrates, such as dry weight loss in lugworms [120], intergenerational developmental responses in copepods [121], anomalous growth delays in juvenile [122] and larval [123] development in sea urchins and ascidians, development parameter alteration in shellfish [124], malformations or dead embryos [105], embryonic development abnormalities [125] in a dose- [120,124,126], time- [127], and size- [128] dependent manner in larvae and adults of different invertebrates. Particularly, the microplastics in the larvae of marine organisms will seriously affect the normal growth of the organism and sometimes microplastics might even cause death, due to their limited abilities to control their internal environment [127]. It was reported that the molting times of the larvae increased significantly in a short period of time after ingesting microparticles [55] and that microparticles had a restrictive effect on their feeding, that is, the microparticles had a sublethal effect on the larvae [55]. Studies have shown that after worms’ ingestion of microplastics, their energy reserves are significantly reduced and particles accumulate in the intestines where they induce inflammation [36].

The effects of microplastics on oxidative stress, inflammatory reactions and metabolic disorders of marine animals were studied. For example, the accumulation of MPs may result in inflammation, lipid accumulation and energy metabolism in fish [128], while oxidative stress and enzyme activity reductions occur in crabs [129].

The adverse impact of microplastic on the reproduction in marine animals, such as egg production [130], fecundity [121], fertilization rates [125], oocyte number [127], population size [130,131] and population growth rate [131] were assessed with significant dose-dependent [130] and distinct size-dependent effects [98,107] being observed in marine invertebrates studies.

At the cellular level, exposure marine animals to MPs induced comprehensive cellular responses. Microplastics could significantly down-regulate histone 3 gene expression [130], and up-regulate Abcb1, cas-8 [132], sod, gpx, idp, pk [133] gene expression. Besides, the activity of phagocytes and mitochondria is significantly increased, and the proportion of oxy radical and immune cells is also up-regulated [134].

#### 3.4.2. Joint Toxicity

Due to the high adsorption capacity of microplastics, many hydrophobic pollutants could adsorb and accumulate on microplastics and accompanied by biomagnification (e.g., PAHs, PCBs, nonylphenols, pesticides, dioxins) [150,157]. Studies have shown that millimeter-sized microplastics have no obvious adsorption toxicity, while micron-sized or even nanosized microplastics have a relatively strong ability to absorb pollutants [131]. For heavy metal pollutants, 32–40 μm plastic particles exposed to heavy metals induce oxidative stress in fish and stimulate their innate immunity [158]. As for organic pollutants, there are studies that have shown that 50 nm plastic particles exposed to PAHs are obviously toxic to aquatic zooplankton and cause significant chemical damage [159]. The biological amplification of organic pollutants becomes higher because plastics reduce the metabolism of pollutants, and the combined toxicity presents an additive effect [160].

In addition to the original monomer, many microplastic products also contain a variety of additives, such as flame retardants, plasticizers, dyes and antioxidants, which make microplastics display joint toxicity with the additives [157,161].

The accumulation and biomagnification of microplastics and their surface-adsorbed pollutants need to be further studied. The joint toxicity may pose a persistent threat to marine ecosystems, due to the durability of microplastics and toxic chemicals [17,162]. Because the toxicity mechanism of microplastics is not fully clear, understanding toxic effects caused by microplastics is important to assess their environmental impacts.

## 4. Bacteria for Degradation of Marine MPs

### 4.1. Bacteria Colonizing Microplastics

Some studies highlight the differences between the bacteria living on organic particles with seawater [166], on microplastics and in a free state [167]. The bacterial community that settles on the surfaces of marine microplastic is significantly different from that in surrounding middle and upper waters or other particle types [166]. If these bacteria have been established enzymatic mechanism for degrading plastic, they would be of particular interest for bioremediation and bioengineering.

Studies show that some bacterial groups such as the phyla *Bacteroidetes*, *Proteobacteria*, *Cyanobacteria* and *Firmicutes* appear to colonize microplastics more often than others, indicating that the specific taxonomic bacteria consider microplastics as a beneficially ecological niche and a potential metabolic adaptation to the material (e.g., attachment, additive resistance, chemotaxis, and degradation). Similar taxa belonging to Bacteroidetes and Proteobacteria seem to be shared by the core bacteria of the seafloor and subsurface plastisphere share, and some photoautotrophic bacteria dominated the sub-surface communities [168,169].

### 4.2. Plastisphere Served as a New Niche for Marine Environment

Recently, the first study using the modern technology of large-scale DNA sequencing gave a detailed image of the microbial communities that inhabit microplastics [128]. Debris is usually described by the term “plastisphere” in marine biology research [169], they serve as various habitats for microbial colonies in aquatic environments besides accumulating organic pollutants [168,169,170,171].

Based on morphological data and DNA sequencing technology, the factors that drive the composition of plastisphere are complex and comprehensive. In addition to the main factors, season and surrounding environment, polymer type, surface feature, and size also affected the diversity and abundance of the colonizing bacterial groups [168,172]. For example, studies highlighted significant differences in microbiota communities on microplastics from the two different oceans, and the diversity of bacteria living in water columns and bacteria attached to microplastic debris [173]. Studies show that plastic surfaces could be rapidly colonized by heterotrophic bacteria, which can survive longer than in the surrounding aquatic environments [174].

### 4.3. Biodegradation of Bacteria in Marine Environment

Microbial biodegradation is a process in which microbial communities (bacteria, actinomycetes and fungi) use organic matter as a carbon source to metabolize, resulting in a transformation from organic carbon to biogas and biomass [175,176]. Generally, the biodegradation process of MPs is proposed to consist of four main basic stages and continuous successive steps: biodeterioration, biofragmentation, assimilation and mineralization [168].

Interest in plastic biodegradation is also growing, and bacteria are considered to be one of the most important ways to solve marine plastic pollution, because of their potential capacity for biodegradation of plastic wastes. *Corynebacterium*, *Arthrobacter*, *Pseudomonas*, *Micrococcus*, *Streptomyces* and *Rhodococcus* are the main bacterial groups in this context, and they can use plastics as sole carbon source under lab conditions [176]. Interestingly, it was discovered that significant differences exist in the diversity, abundance and activity of bacterial and physiochemical characters of plastics between biodegradable and non-biodegradable plastics, indicating the presence of plastic-degrading microbes [177]. Nowadays, there is an increasing number of anecdotal evidence that bacteria can show the capability to degrade ocean plastic pieces [169,172,174] (Table 3).

The factors involved in plastic biodegradability depend not only on the ability of microorganisms but also on the characteristics and surface structure of the material, such as the roughness, electrostatic interactions, topography, hydrophobicity, and free energy [106]. In addition, various environmental factors, such as oxygen level, temperature, humidity, salinity, and limitation of light have an important impact on the biodegradation of plastics [186]. The additives in the polymer could increase the rate of biodegradability. These additives will affect their chemical and thermal sensitivity as well as their ability to absorb ultraviolet light and lead to the loss of stable properties that are more suitable for microbial attachment [187].

The current test standards for assessing plastic biodegradability of marine plastics tend to use to use optical, atomic force and scanning electron microscopy to confirm the results of major tests based on respirometers, since each of them has limitations, and none of these techniques are sufficient by itself [188]. To date, standard guidelines and methods for conducting these experiments have not been established.

Our understanding of metabolic mechanisms of biodegradable marine bacteria and their enzymes is very limited. Furthermore, the biodegradation mechanics of marine plastic debris and its potential impact processes need further research to make full use of its impact.

## 5. Conclusions

The accumulation of microplastics in the marine environment is a serious threat to the health of marine organisms, which may eventually affect the survival of human beings. Therefore, it has attracted extensive attention from society and researchers. Many studies have shown that different bacterial communities colonize microplastics in the marine environment, which has inspired us to investigate the bacterial degradation of marine microplastics. However, until now, we don’t know much about how these bacteria work. The rich diversity and activity of these bacteria indicate their potential in the biogeochemical cycling of plastics, but further research is needed. Contact experiments must be carefully designed to test the ability of these bacteria to react with plastics and adapt to changing marine environments, so it is important to integrate research approaches from multiple disciplines. In order to take full advantage of the influence of bacterial communities on MPs, more controlled experiments are needed to simulate real marine ecosystems. Further studies of bacteria associated with plastic degradation will help develop situ biodegradable methods and materials. According to the current technology and methods, it is impossible to completely remove all the microplastics in the ocean, but we can still try to partially reduce marine microplastic pollution. Bacterial degradation is an appropriate choice for this. While developing methods for degrading plastics, relevant stakeholders such as governments, the public, manufacturers and scientists should pay high attention to the problem of marine microplastics pollution. We should take responsibility and working together to reduce unnecessary plastic production and reduce plastic waste by recycling plastic to tackle increasing MP issues.

## Figures and Tables

**Table 1:** The characterization of MPs in marine ecosystem.

Location	Sample Type	Mesh Size	Concentration	Particle Size	MP Type	Poly Type	Reference
**North African coasts of Mediterranean** **European seas**	surface sediments beach litters	NA	182.66 ± 27.32 649.33 ± 184.02/kg sediment DW	NA	fibers (70%), fragments (21%), pellets (5%), films (2%) and foams (2%)	PE (48%), PP (16%), PET (14%), PS (9%), butyl branham (7%), EPM (3%), TCA (3%)	[102]
**Nordic Seas**	seawater	NA	1.19 ± 0.28 items/L (EGC), 2.43 ± 0.84 items/L (GSG)	0.1–0.5 mm	fiber (76.1%), transparent (76.2%), small microplastics (48.1%)	PA, PE, PET, PMMA, PP, PS,	[103]
**Terra Nova Bay, Antarctica**	macrobenthic species	NA	1.0 items/individual, 0.7 items/mg DW	50 and 100 μm	nylon (86%), polyethylene (5%)	PAA, PARA, PA, PP, PS, PTFE	[104]
**Southern Caspian coastal northern, Iran**	coastal sediment	250–500 µm	25 items/kg 330 items/kg	<2 μm	fiber, fragment, film	PS, PE	[105]
**Banten Bay, Indonesia**	sediment	0.45 µm	mean: 267 ± 98 particles/kg DW, min: 101 particles/kg DW, max: 431particles/kg DW	500 and 1000 μm (>50%)	foam (30.4%), fragments (26.5%), granules (24.4%), and fibers (18.7%)	Cellophane, PS	[106]
**Caspian Sea**	surface waters sediments	50 μm 0.3 mm	34,490 particles/km^2^, 210 particles/kg	1–4.75 mm in surface water (68%) and coastal sediments (50%)	fragment (38%), styrofoam (31%), film (20%), lines (9%) (surface water) styrofoam (35%), fragment (31%) (sediment)	PE, PP, PS	[107]
**Boknafjord, Norway**	sediments	10–250 μm	11 to 140 μg/kg DW	40–100 μm	NA	PE: 32.3–139.2 μg/kg PVC: 9–120 μg/kg PET: 12–136.5 μg/kg PP: 10–78.4 μg/kg PA: 16–73.1 μg/kg	[108]
**Kingston Harbour**	surface waters	335 μm	mean: 674.13 particles/km^2^, min: 5.73 particles/m^3^, max: 2697 particles/m^3^	1–2.5 mm	fragment	PE, PP	[109]
**Northwestern Pacific Ocean**	surface waters	330 μm	mean: 1.0 × 10^4^ items/km^2^ (6.4 × 10^2^–4.2 × 10^4^ items/km^2^)	0.5–1.0 mm (50%), 1–2.5 mm (29.8%), 2.5–5.0 mm (17.6%)	granules (39.7%) sheets (26.7%), films (24.7%), and lines (8.9%)	PE (57.8%), PP (36.0%), PA (3.4%)	[110]
**Qinzhou Bay, China**	sediment	5 mm	15–12,852 items/kg	0.16–5.0 mm	fragment (94%), sphere (5.2%), fiber (0.5%)	PS, PP, PE, oxidized PE, LDPE	[111]
**Irish Continental shelf, Atlantic**	sediment bottom water	250 μm	Max: 0.5 cm	250 μm– 5 mm	fibers (85%) fragments (15%)	23% PA, 11%PET, 3% PP, 2% acrylic	[31]
**Deep–sea of Mid–Atlantic and Indian Ocean**	organisms		found inside oral or stomach area	NA	100% Fibers	Modified acrylic, PP, PET, viscose, acrylic	[32]
**Polar waters, Arctic**	surface and subsurface water	333 μm	NA	Surface: 0.34/m^3^, Subsurface: 2.86/m^3^	fibers (95%) fragments (4.9%)	30% Rayon 15% PET 15% PA 5% PE	[112]
**NE Pacific Ocean**	subsurface seawater	7.8 × 7.5 mm	8–9200 particles/m^3^	Mean: 606 ± 221 μm (62–5000 μm)	fibres, fragments	NA	[113]
**Beach** **Continental shelf (Belgium)**	sediment	38 μm	92.8/kg 97.2/kg	38 μm–1 mm	fibers (59%), granules (25%)	PP, PA, PVA	[30]
**Beach** **Continental shelf (UK)**	sediment		0.4/50 mL 5.6/50 mL	~20 μm	fibers	9 polymers	[64]

PE: polyethylene; PP: polypropylene; PS: polystyrene; EPM: ethylene propylene diene; TCA: tricellulose acetate; PA: polyamide; PET: polyethylene terephthalate; PMMA: poly methyl methacrylate; PAA: poly (acrylic acid); PARA: polyaryl amide; PTFE: polytetrafluoroethylene; LDPE: low-density polyethylene; PVC: polyvinyl chloride. NA: Not available.

**Table 2:** Effects of microplastics and nanoplastics on marine organisms

Phyla	Species	Development	MP Size	Adsorption	MP Types	Negative Effects	References
Bacillariophyta	*Chaetoceros neogracile*	spore, adult	50 μm	NA	PS	Particles decrease chlorophyll content, esterase activity, cell growth and photosynthetic efficiency of diatoms.	[163]
Aschelminthes	*Brachionus koreanus*	adult	0.05, 0.5, 6 μm	NA	PS	Inhibition of multiple resistance to p-glycoproteins and multidrug resistant proteins leads to increased toxicity and oxidative stress damage to membrane lipids.	[164]
Mollusca	*Crassostrea gigas*	embryo, larva, adult	50 μm	NA	PS	Particles reduce fertilization rate and development ability of embryo and larva.	[48]
	*Mytilus galloprovincialis*	larva	140 ± 34.6 nm	Cbz	PS	Increased total oxidant status of digestive glands, influence neurotransmission, genotoxicity and lipid peroxidation.	[45]
		adult	0.1–1 mm	pyrene	PE, PS	Alter immune response, lysosomal compartment, peroxisome, antioxidant system, and neurotoxic effects	[46]
Arthropoda	*Artemia franciscana*	larva	40, 50 μm	NA	PS	Impairment of feeding ability, behavioral ability and physiological conditions.	[55]
	*Calanus finmarchicus*	adult	particles: 10–30 μm fibers: 10 × 30 μm	NA	PA	Alter predation behavior, reduce fat storage, and affect growth and development.	[47]
Chordata	*Danio rerio*	embryo	average: 398 ± 54 μm minimum: 10 ± 2 μm	NA	PE	Produce cell death and affect energy metabolism.	[49]
			50, 200, 500 μm	Au	PS	Oxidative stress and inflammation reaction.	[50]
			44 nm	PAHs	PS	Energy metabolism.	[51]
		larva	25 μm	NA	PS	Glucodermatin receptors disrupt glucose homeostasis, leading to abnormal larval activity.	[52]
			44 nm	PAHs	PS	Energy metabolism.	[51]
		adult	25 μm	Cu	PS	Inflammatory reaction.	[53]
			50 μm	BPA	PS	Neurotoxicity.	[54]
	*Fish cell lines* (*SAF-1*, *DLB-1*)	/	100 nm	NA	PS	Change the activity of superoxide dismutase and Glutathione S-transferase and the toxicity of drugs.	[165]

PS: polystyrene; PE: polyethylene; LDPE: low-density polyethylene; HDPE: high-density polyethylene; PA: polyamide; PP: polypropylene; PUR: polyurethane; PET: polyethylene terephthalate; PVC: polyvinyl chloride. PAHs: polycyclic Aromatic Hydrocarbons; BPA: bisphenol A; Cbz: carbamazepine. NA: Not available.

**Table 3:** Outstanding plastic-degrading bacteria in existing research.

Plastic Types	Year	Strains	Source	Plastic Forms	Weight Loss	Principle	References
PS	2015	*Exiguobacterium* sp. *YT2*	Intestines of *Tenebrio molitor*	sheet	(7.4% ± 0.4%)/60 days	NA	[178,179]
LDPE	2014	*Bacillus* sp. *YP1*	Intestines of *Plodia interpunctella Hübner*	film	(10.7% ± 0.2%)/60 days	NA	[180]
HDPE	2010	*GMB7*	Plastic waste landfill in Mannar, India	film	15%/30 days	NA	[181]
PA	2000	*Flavobacterium* sp. *KI72*	NA	NA	NA	Hydrolysis of polymer hydrolases	[177]
PP	None						
PUR	1995	*Comamonas acidovorans TB-35*	Soil	film	100%/7 days	Hydrolysis of esterase encoded by gene *PudA*	[182,183]
	2014	*Pseudomonas putida A12*	Soil	emulsion	92%/4 days	Hydrolysis of a 45 kDa esterase	[184]
	2017	*Bacillus* sp. *S10-2*	Spacecraft	emulsion, film	19%/60 days	Hydrolysis of esterase	[185]
PET	2011	*Bacillus subtilis*	Laboratory	film	NA	Hydrolysis of p-nitrobenzylesterase	[45]
PVC	None						

PS: polystyrene; PE: polyethylene; LDPE: low-density polyethylene; HDPE: high-density polyethylene; PA: polyamide; PP: polypropylene; PUR: polyurethane; PET: polyethylene terephthalate; PVC: polyvinyl chloride. NA: not available.
